# Supplementary material for: A geometric criterion links HIV-1 capsid topography to its biophysical properties and function
Source: Nat Commun. 2026 Jun 15;17:7536. doi: 10.1038/s41467-026-74109-6 (PMC13408807; doi:10.1038/s41467-026-74109-6)
Supplement: Supplementary file 2 — Description Of Additional Supplementary Files [file 41467_2026_74109_MOESM2_ESM.pdf]

Description of Additional supplementary files

Supplementary Movie 1 – Molecular frustration at the reference dimer interface

Description: The reference dimer interface (PDB: 2KOD). Residue pairs involved in H9 helix interactions are shown as solid lines (direct contacts) or dashed lines (water-mediated contacts), coloured red for highly frustrated, green for minimally frustrated, and grey for neutral interactions. This movie corresponds to Fig. 4a.

Supplementary Movie 2 – Molecular frustration at the lowest frustrated dimer interface in capsid vlp23

Description: Representative dimer interfaces across two hexamers in vlp23 with the lowest local frustration states. This movie corresponds to Fig. 4b.

Supplementary Movie 3 – Molecular frustration at the highest frustrated dimer interface in capsid vlp23

Description: Representative dimer interfaces across two hexamers in vlp23 with the highest local frustration states. This movie corresponds to Fig. 4c.

Supplementary Movie 4 – Topological patterns on the surface of capsid vlp23

Description: Pairs of neighbouring threefold axes are colour-coded by geometric index orientation: grey (type 1), purple (type 2), dark blue (type 3), orange (type 4), and red (type 5) as defined in Fig. 6d. This movie corresponds to Fig. 6e-f (right).

Supplementary Movie 5 – Predicted CypA binding sites in a CA tubular assembly

Description: CypAs (light purple) are mapped onto the predicted binding sites on a CA tubular structure (PDB: 6X63) by aligning the CypA-binding monomer from the complex (PDB: 6ZDJ; Supplementary Fig. 10e) to the corresponding CypA-binding monomers on the tube. This movie corresponds to Fig. 7c (right).

Supplementary Movie 6 – Molecular frustration before CypA binding

Description: Apo CA complex prior to CypA binding, with three regions from different hexamers highlighted in distinct colours. Molecular frustration at the binding region and dimer interfaces is shown. This movie corresponds to Fig. 8c.

Supplementary Movie 7 – Molecular frustration after CypA binding

Description: The CypA-CA complex with CypA shown in hot pink. Molecular frustration at the binding region and dimer interfaces is shown. This movie corresponds to Fig. 8d.
