# Supplementary material for: A geometric criterion links HIV-1 capsid topography to its biophysical properties and function
Source: Nat Commun. 2026 Jun 15;17:7536. doi: 10.1038/s41467-026-74109-6 (PMC13408807; doi:10.1038/s41467-026-74109-6)
Supplement: Supplementary file 11 — Transparent Peer Review file [file 41467_2026_74109_MOESM11_ESM.pdf]

nature portfolio

Peer Review File

A geometric criterion links HIV-1 capsid topography to its biophysical properties and function

Corresponding Author: Mr Wenhan Li

This file contains all reviewer reports in order by version, followed by all author rebuttals in order by version.

Version 0:

Reviewer comments:

Reviewer #1

(Remarks to the Author)
The authors introduce a geometric framework to characterize local lattice organization in the HIV-1 capsid and relate this geometry to curvature, molecular frustration, and cofactor binding. By defining a local geometric index based on characteristic triangles at threefold junctions, the authors move beyond idealized fullerene representations and argue that a data-derived lattice more faithfully captures capsid topography. The work is very interesting, technically solid, and connects geometric descriptors to biophysical observables in a novel way.

The manuscript will be of interest to researchers studying viral capsid assembly, mechanics, and capsid-targeting therapeutics. I recommend publication after minor clarification. The questions below are intended to clarify conceptual points and do not require additional analyses.

1. When referring to “triangular gaps between the capsomers” in the data-derived lattice, do the authors mean the small triangles illustrated on the right side of Fig. 1d? Could the authors clarify whether these represent geometric mismatch relative to an idealized lattice or physical gaps between proteins?

2. The authors note that hexagons in fullerene cones are typically distorted as part of a surface lattice. In earlier models and growth scenarios, however, hexamers remain topologically equivalent to a flat hexagonal sheet except in the immediate vicinity of pentamers and along seam lines, where curvature and lattice mismatch are concentrated. Could the authors clarify whether they observe distortion of hexamers throughout the entire lattice in their data-derived models, or whether such distortions are likewise localized primarily near pentamers and seam regions? Some clarification on why hexamers deviate from a regular hexagonal sheet, which is often locally energetically favorable, and whether such deviations occur globally or are enhanced near pentamers or seam regions would help the reader.

3. In Ref. 28, triangular gaps arise specifically along the seam as a consequence of lattice mismatch, while the remainder of the capsid is well described by a hexameric lattice except near pentamers. Do the authors observe triangular gaps or analogous geometric features away from seam regions in the present data-derived lattice, or are such features likewise localized primarily near seams and/or pentamers?

4. The authors state that “typically, two to three seams appear in the bulk of each cone, joining adjacent sheets.” In Ref. 28, cones typically exhibit a single seam, and some cones grow without seams altogether. Can the authors’ model justify the presence of multiple seams energetically, given that seams are expected to carry an energetic cost? In addition, experimental work (e.g., Ganser and collaborators) has reported cones with different pentamer distributions (e.g., 5–7, 3–9, ….). Have the authors considered these scenarios?

5. Given that seam-like features can arise transiently or persist in cylindrical assemblies depending on growth pathways (e.g., Ref. 28), it would be helpful if the authors clarified whether the absence of seams in their cylindrical models reflects the specific construction and relaxation protocols used, and whether their geometric framework would be able to detect seam-like features in kinetically assembled cylinders.

6. Instead of (or in addition to) the length of the shortest edge of the characteristic triangle, did the authors consider alternative geometric measures, such as a strain-like quantity directly related to elastic moduli (e.g., Young’s modulus)?

Such a metric might offer a more direct mechanical interpretation and could be worth discussing, even if only briefly.

7. When discussing curvature, do the authors primarily refer to angles defined between characteristic triangles, or to dihedral angles between neighboring capsomers? In Ref. 28, differences between cones and cylinders arise from differences in dihedral angles, and more recent kinetic studies of icosahedral assembly (Science Advances, 11, eady7241 (2025) have shown that changes in dihedral angles can alter assembly pathways (e.g., T=3 vs T=4). Clarifying how curvature is defined and interpreted here would help connect the present framework to these earlier results.

(Remarks on code availability)

Reviewer #2

(Remarks to the Author)
Twarock Revisión:

Li and coworkers present a study arguing that the geometry of the HIV-1 capsid is more accurately captured by a data-derived pseudo-tiling arrangement of hexamers and pentamers, rather than by the idealized fullerene lattice traditionally used to describe it.

To support this hypothesis, the authors introduce a local geometric criterion to quantify deviations from an ideal fullerene lattice and show that these deviations correlate with specific biophysical properties of the capsid. They further suggest that such geometric deviations, and their associated biophysical features, provide insight into functional aspects of the capsid such as cofactor binding.

I consider the article is very interesting, the results are novel and I really enjoyed learning more about viruses and their properties while revising it.

Minor Comments:

Introduction:
In line 41 it says that there are 200 hexamers and 12 pentamers and that in many spherical viral shells there are deviations from the idealised lattices. In lines 60/61 it says that there is an imperfect alignment between adjacent hexamers / pentamers. I kind of understand the flow, but it is not clear that this observation comes from the atomic model derived from experiments in Fig1A, neither the panels are referenced but just Fig1 in general. I would ask authors to clarify so it is clearer and to reference individual panels in Fig1 to provide examples of each case.

Fig1a is mentioned later when they introduce that CryoEM has advanced… in my opinion it feels like the introduction comes forward and backward in the logical flow. It is understandable but a bit confusing.

Line 68. I would write “Here in this work, we demonstrate…” to make it clear that it is this work and not past work from the authors. Also the word demonstrate sounds a bit too strong in this context.

You mention the hexamers and pentamers but no protein structures are shown in detail. I would like to maybe see them as an extra column in Fig1? 1 Hexamer and 1 Pentamer arrangement? Only the Hexamer appears in Figs 3 and 6.

Fig2d legend: jointed -> joint.

Fig3: There are two lattices per panel. Are they rotations I guess (as they mention opposite sites of the structures in line 218), but not sure. Not described in the legend.

REsults:

Fig2: It is not clear to me from the text nor from the figure legend what are the 3 structures in panel d. Are they the same structures as in Fig1a with the geometric indexes and seam lines on top or are these rotations of the fullerene cone (as mentioned in the legend? the other structures/models are not mentioned and from there my confusion). In general, labels in the panels are easier to follow rather than having to read the legends.

Line 219 the authors use “demonstrate” again. This is maybe personal but to show something is different from demonstrating.

Major Comments:

The authors analyse the binding of CypA and LEN. As CypA is a protein, can the authors analyse frustration changes upon binding between the lattice proteins and the CypA? The authors also say that binding at the CypA site induces local increase of the curvature whereas the opposite effect is observed when binding at the FG-motif pocket. Does it correlate with frustration differences in those sites and the curvature change?

In the section “Correlation between capsid topography and molecular frustration at dimer interfaces”

The authors say “we extended the concept of molecular frustration from the Protein Frustratometer…”. I disagree with this wording. What the authors are doing is to calculate the average frustration per contact involving the h9 helices at the interfaces. That is not, in my opinion, a conceptual extension but an application. The authors are analysing the frustration patterns as calculated by frustratometer at the interfaces. I suggest rewriting this part.

In this same section, the authors refer to frustration “levels”, whereas typically we refer to frustration “states”. Also, the authors change the frustration index sign which is confusing regarding how frustration indexes are interpreted in the literature where high frustration is denoted as negative Z-scores compared to the decoys and minimal frustration as positive. Changing the sign of the indexes could confuse readers and I do not see this is needed to make the analysis and interpretations the authors are making.

In the same section the authors could cite the papers where frustration is correlated with the functional location of binding sites (ferreiro et al pnas 2007, parra et al plos comp biol 2015) and allosterism (ferreiro et al pnas 2011).

The authors also mention: “In the original approach, the frustration level of a residue is derived from the proportion…”. This statement is inaccurate and may mislead readers unfamiliar with the Frustratometer methodology.. Frustration states of residues/contacts are based on the comparison of the total energy change between the native protein and the decoys. There is another secondary analysis Frustratometer returns that is the proportion of contacts classified in each frustration state so the contact frustration indexes (configurational and mutational) can be assessed at single residues, which complements the single residue frustration index that can mask bimodal distributions of states. Such analysis counts the proportion of contacts of each state in a sphere of 5 Angstroms. In that sense, I guess we could say their analysis is somehow analogous. Still, I think the authors should rephrase to be precise about these issues.

The results shown in Fig4 and movies 1, 2 and 3 are very interesting. In frustration analysis we have 3 types of contacts: short, long and water mediated. We usually show water mediated interactions in dashed to differentiate them from the short and long. In the movies everything is shown in dashed lines (I guess for simplicity) so I cannot differentiate the water mediated from the non water mediated contacts. The different contact types (the classification is available in the frustratometer output) use slightly different hamiltonians based on distance and solvent accessibility. It would be interesting to observe if the longer distances between interacting proteins correlate with solvent accessibility changes and changes in overall frustration. If yes, this may point out that the geometric index and changes in curvature modulate function by changing solvation at the interfaces which is translated in frustration differences between models and so on.

In Fig4f I got confused. What is the frustration “level” that is being plotted? Is their averaged frustration value per interface contact involving h9 helices and the number of contacts being considered in such calculation? Basically, when there are less contacts in the interface they are also more highly frustrated on average? (I had to see these multiple times as the change in sign made me conclude the opposite, see previous comments about consistency with previous frustration analysis) In that panel, I would be a bit cautious. The number of interface contacts (y axis) ranges from ~200 to ~260, while the mean frustration changes (x-axis) by ~0.5 units and remains largely within the neutral regime. While the trend is clear, additional analysis (e.g., proportions of highly/minimally frustrated contacts) would strengthen the interpretation. The effect is there, I am not doubting it, but to make it stronger I think a bit of analysis of proportions would help. In our experience averaging always takes one to the neutral region. That is why using discrete states as proportions usually helps. The authors could analyse to put on the x axis the proportion of interface contacts being actually red, gray or green. Are more dense interfaces (shorter gap) harbouring more red contacts? I think it might be true that interfaces that are more solvated lead to less contacts (because of distance) and the ones that remain as they are water mediated are more frustrated. When distances become shorter, they become energetically more optimized. But the authors should analyse this more carefully and see if this is true. From panels a,b,c I am not sure if there are more highly frustrated interactions but more neutral instead when the gap is larger. When the gap becomes shorter there are more minimally frustrated contacts. This could be enough to explain the effect, very interesting if true. It would be useful to assess whether the co-localization of frustration and seam lines is robust to alternative frustration aggregation schemes (e.g., median frustration, weighted by contact area, or excluding water-mediated contacts), as averaging may mask heterogeneous distributions.

I am not sure what the authors want to say in lines 263-264: “Strikingly, ..”.

The co-location of frustration with the seam line is a very compelling result that could explain the functional modulation of the capsid.

All over the manuscript, there is no consistent mention to the models. Sometimes they are named as idealised, data derived, etc.. whereas in other cases are mentioned by their pdb ids or other nomenclature. I think the authors have to refer to the different models and lattices in a consistent way so it is easy to keep the logic through sections. It is especially difficult when PDB IDs are used. The authors are really familiar with them but for non related readers those IDs mean nothing and it is difficult to connect/remember which one is which.

I wonder if authors could make a big matrix of pairwise correlations of: number of interface contacts, averaged frustration per interface contacts, geometric index, curvature factors phi1 and phi2, etc. Because are the values in Fig6c correlated with number of contacts and frustration? If yes, how would it be interpreted? I guess this is related to the sentence in lines 373-375. To observe those correlations would strengthen the claim.

Line 377: “universal”. Sounds too strong. Do you mean it could be used to describe any other capsid from any other virus? I would use such a word if a very large set of case studies would be analysed with this.

In Fig7 the authors show binding of CypA on top of the lattice arrangements. Did the authors quantify frustration in the CypA-bound state and CypA and compare if the complex leads to less frustration when quantified in the complex vs in the monomers alone? Release of frustration upon binding, even with this artificial way would be a good support to their results.

Methods:

By checking the supplementary material I see that the authors have used the configurational frustration index. Which is good as it correlates better with protein-protein interfaces. However, the authors do not mention that the configurational index is used. Please do so readers know.

The authors say: “Local energetic frustration was quantified based on previous work”. I understand that the authors did not code their own algorithm to calculate frustration but used Frustratometer. So I think the correct wording would be “Local energetic frustration was quantified using the Frustratometer tool (citations). The configurational frustration index was used as it has been shown that it better correlates with protein-protein binding sites (Ferreiro et al pnas 2007, parra et al plos comp biol 2015).

Summary of my revision:

I think the article by Li et al presents a very interesting way to link local geometries to higher order arrangements that in turn lead to specific frustration patterns in the lattices that may lead functional processes like the binding of co-factors.

The text is sometimes difficult to read due to vague or inaccurate phrasing and inconsistent use of terminology (at least in the case of frustration). Figures not having labels force readers to go back and forth between them and legends' difficult interpretation. Specifically, the way they refer to the different lattice geometries took me a lot of time to make sense of the data.

That being said, I do think that the main results are supported by their analysis. I have proposed some complementary analysis that in my opinion would strengthen their claims.

To me the most interesting part is that according to their data, the distorted lattice geometry that has more frustrated interactions on average (and broader gaps) would not be my first choice between models because one would expect protein-protein interactions to be minimally frustrated in a well formed capsid. The fact that the model supported by the data has the border gap with more frustration suggests a functional requirement, maybe to bind the co-factors. That is not discussed as such in the discussion or anywhere in the text.. maybe it is not true, but the authors have the data to prove it and if true, that is REALLY interesting, at least to me.

It is also surprising to me that the authors only briefly mention their work in reference 40 that has nicely shown how local frustration can help orchestrate virus assemblies.

(Remarks on code availability)

Reviewer #3

(Remarks to the Author)
In the paper by Li et al, they define a new geometric metric, referred to as the geometric index, that reports a length scale on each 3-fold axis within a defined lattice. The key observation is that this index varies by lattice assigned to a structure: fullerene vs kagome vs data-derived, and for the data-derived lattices, it is a good predictor of lattice energy and curvature due to the correlations. The index also correlates with binding sites for small molecules.

The metric certainly seems useful, and the figures are generally clear about the geometric aspects, although several of the figure subplots with numerical data are hard to read. It is unexpected that the definition of the lattice topology (e.g. fullerene vs data-derived) which primarily differ in what angle the hexagons adopt relative to one another, is more or less informative about the lattice properties. The comparison of the topologies is also interesting, that drawing boundaries between hexagons in distinct ways can be more or less informative about the lattice properties. Frustration between dimers, for example, is correlated with the data-derived lattice seams. Overall, the geometric metric, if it is easy enough to calculate from an experimental structure, seems to be an informative way to visualize the structure and assess where frustration may exist in the lattice. It is harder to tell how predictive it will be.

A limitation is that the results of the geometric index seem to be correlative, rather than causative or truly predictive. The authors did not use the index to test a hypothesis about is predictive power, by, for example, designing a structure to have specific properties, whether of energetics, frustration, or binding. It is also not clear to me how many distinct HIV capsid lattices were actually evaluated, and therefore how robust the trends are (what is N?) I think it is 3, vlp23 and two PDB structures, but this is not clearly stated.
Major comments:

1. What are the C524, etc, datasets? Is this one single capsid lattice? From the SI it looks like maybe 3 lattices were studied? They mention Carbon atoms repeatedly, but the subunits of the capsid lattice are complete proteins, so I don't understand this. Are the 654 fullerene structures each a different experimental lattice? Why are there such specific numbers (325, 452) of vertices? What is vlp23, a single structure?
2. In Figure 2f, for geometric index vs energy, I'm not quite sure what energy is being reported—the range is very small, from -9.685 to -9.702, is this significant? What is each dot in the plot from?
3. For Figure 5, they are comparing lattices that were constructed using distinct protocols during the cryoEM reconstruction. I am not sure what to conclude. Are the authors suggesting that the atomic structures fit to the lattices are more accurate depending on which protocol is used, or that the properties of the lattice will be different depending on how the atomic structure is fit to the cryoEM data?
4. Following this, the authors mention that the geometric index can report on assembly pathways. This would be interesting, but is fairly indirect given that the data are based on reconstruction methods rather than dynamic assembly trajectories.
5. The analysis of the CypA binding and the LEN binding is a bit hard to follow. Binding of the cofactors are reported as correlated with the geometric index, but I don't see this from Fig 7 data. I can't tell what is analysis of the known data, and what is the prediction.
6. The step of showing how the geometric index could be used in practice to design something with specific properties or select for stronger or weaker binding of co-factors is not pursued. This leaves the study as more speculative than truly demonstrative of its utility.

(Remarks on code availability)

Version 1:

Reviewer comments:

Reviewer #1

(Remarks to the Author)
The authors have revised the manuscript in response to the comments from the other referees and myself. I therefore recommend the paper for publication.

(Remarks on code availability)

Reviewer #2

(Remarks to the Author)
I am pleased that the authors have taken my suggestions seriously and have made a careful and thorough revision of the manuscript. I appreciate the clear effort made to address the majority of my comments, both in terms of improving the clarity of the presentation and, importantly, by incorporating additional analyses which I consider have made their article stronger. In particular, the new results linking cofactor binding, the curvature changes, and molecular frustration (Fig. 8 and associated supplementary material) significantly strengthen the manuscript and provide a more compelling connection between geometry and biophysical function.
I also note that the authors have corrected important technical aspects regarding the use and interpretation of molecular frustration (terminology, sign conventions, and methodological descriptions), which improves the rigor and accessibility of the work. The improvements in figures and labeling have also made the manuscript easier to follow.
That said, I have the following minor points and curiosities from my side:
The word demonstrate still remains in the abstract. I checked the authors have modified it in the main text.
Page 11. When the authors explain that they used the configurational frustration index, they use the word metric instead of index. The frustration index is a z-score and therefore not a metric which is a mathematical concept that satisfies very specific constraints. The correct word is index, not metric.
In page 17, they also use the word metric to refer to the curvature which would be more accurately referred to as "measures", "scores" or any other word that better reflects what that quantity is.
Page 19. They also use the word metric to refer to the geometric "index".
The analysis of frustration changes in the interfaces with CypA and CPSF6 is interesting. The change that is most noticeable is the delta in binding of CypA followed by the delta in the binding of CPSF6. For the interfaces, maybe the averaging is masking some interesting results. First, it would be good to add the number of considered residues both at the binding sites and at the interfaces in both cases which I can kind of quantify from supp figs 16 and 17(add to Table 1). But also, you could include the mean proportion of virtual particles of each residue (you can find that data in the 5Adens output for the configurational index) that tells you the proportion of each frustration class in a 5 Angstroms sphere from each residue (e.g. the ones at the binding and interface residues in each case). This may unmask some interesting patterns (similar to the fig2 in this article from the last author: https://www.cell.com/trends/microbiology/fulltext/S0966-842X(23)00200-7). The number differences in the table are relativelly small, in my opinion, both in the neutral range.. I would not overinterpret them unless a dissection as explained before throws some more meaningful result. In Fig 16 I do not see a big difference at the interface of CypA. I do see a bigger difference for CPSF6. Is the binding of these proteins to the capsid triggering some sort of allosteric response? I think that is a very nice hypothesis that the authors have written in that section although I would say the allosteric effect is larger in CPSF6, if true :)
In the same page the authors say "while these observations highlight opposing geometric and frustration responses at the

two primary binding sites". Again, the changes are small and both remain in the neutral region of frustration and also are measured in static complexes. I would not overinterpret the effect at the interfaces as "opposing" as they are energetically in the same class of frustration both after and before the binding. In addition, the authors could analyse the residues of each group (binding and interfaces) individually. The averaging may be masking some energetic signature because not all residues behave the same way. As assembly and disassembly are reversible processes when some residues reduce frustration after binding, some others may increase it (see https://www.nature.com/articles/s41586-025-09549-z) and vice versa. Therefore, the authors can analyse residues individually to see which are the residues that have the highest change and potentially drive the process while the others just follow or prone the complex to dissociation once bound.
Fig 8g and h. Unfortunately, the color of the protein structures (red and yellow) is very similar to the frustration lines (red and green) and it becomes difficult to see anything.I would play around with the protein structure colors to make it less difficult to observe.
In summary, I think that the article is greatly improved and clarified. I just would like the authors to evaluate every instance where they use the words demonstrate and metric. I think that in most cases, if not all, the use of these terms is inaccurate. The message is clear, but accurate wording is clear and nicer.

Congratulations for such nice work that enriches the applicability of frustration analysis to complex systems. I do not consider it necessary to review the manuscript again.

(Remarks on code availability)

Reviewer #3

(Remarks to the Author)
The authors have responded to my comments, the figures and the text are more clear, as are the predictive applications.

(Remarks on code availability)
The Zenodo repos do not appear to be active, I cannot find either doi, I also searched by paper title and did not find them.

Open Access This Peer Review File is licensed under a Creative Commons Attribution 4.0 International License, which permits use, sharing, adaptation, distribution and reproduction in any medium or format, as long as you give appropriate credit to the original author(s) and the source, provide a link to the Creative Commons license, and indicate if changes were made.
In cases where reviewers are anonymous, credit should be given to 'Anonymous Referee' and the source.
The images or other third party material in this Peer Review File are included in the article's Creative Commons license, unless indicated otherwise in a credit line to the material. If material is not included in the article's Creative Commons license and your intended use is not permitted by statutory regulation or exceeds the permitted use, you will need to obtain permission directly from the copyright holder.
To view a copy of this license, visit https://creativecommons.org/licenses/by/4.0/

REVIEWER COMMENTS

Reviewer #1 (Remarks to the Author):

The authors introduce a geometric framework to characterize local lattice organization in the HIV-1 capsid and relate this geometry to curvature, molecular frustration, and cofactor binding. By defining a local geometric index based on characteristic triangles at threefold junctions, the authors move beyond idealized fullerene representations and argue that a data-derived lattice more faithfully captures capsid topography. The work is very interesting, technically solid, and connects geometric descriptors to biophysical observables in a novel way.

The manuscript will be of interest to researchers studying viral capsid assembly, mechanics, and capsid-targeting therapeutics. I recommend publication after minor clarification. The questions below are intended to clarify conceptual points and do not require additional analyses.

1. When referring to “triangular gaps between the capsomers” in the data-derived lattice, do the authors mean the small triangles illustrated on the right side of Fig. 1d? Could the authors clarify whether these represent geometric mismatch relative to an idealized lattice or physical gaps between proteins?

Thank you for your helpful questions. We have revised **Fig. 1** to more clearly illustrate the geometric organisation, with the protein capsomers superimposed on the lattice representations.

The “gaps between the capsomers” refer to the regions between neighbouring hexagons (and pentagons) shown in Fig. 1f-i. **These features arise from geometric mismatch in the gyrated lattice relative to an idealised lattice**. Specifically, local rotations and positional adjustments of hexamers and pentamers – arising from the formation of dimer interfaces (red lines between capsomers in Fig. 1f-i) – lead to an imperfect alignment of the capsomers boundaries.

Importantly, **these gaps are not physical voids between proteins**: the corresponding interfaces are fully occupied by protein-protein contacts in the atomistic structures (Fig. 1h-i). Moreover, such mismatched regions are not simply triangular, but occur at both threefold axes and twofold axes. We have clarified this distinction in the revised manuscript to avoid ambiguity.

2. The authors note that hexagons in fullerene cones are typically distorted as part of a surface lattice. In earlier models and growth scenarios, however, hexamers remain topologically equivalent to a flat hexagonal sheet except in the immediate vicinity of pentamers and along seam lines, where curvature and lattice mismatch are concentrated. Could the authors clarify whether they observe distortion of hexamers throughout the entire lattice in their data-derived models, or whether such distortions are likewise localized primarily near pentamers and seam regions? Some clarification on why hexamers deviate from a regular hexagonal sheet, which is often locally energetically favorable, and whether such deviations occur globally or are enhanced near pentamers or seam regions would help the reader.

Thank you for your insightful questions. In this work, the data-derived lattices are constructed directly from reconstructed atomistic capsid structures, and the extent of hexamer distortion depends on the specific reconstruction protocol used (see Supplementary Fig. 9).

For the *vlp23* capsid, the complete structure was first assembled by fitting capsomers into a cryo-EM density map along a Hamiltonian path, followed by molecular dynamics (MD) relaxation of the entire capsid. In this case, the resulting hexagonal and pentagonal representations remain close to regular across the surface.

By contrast, the capsids corresponding to PDB structures 3J3Y and 3J3Q were generated using a different workflow, in which MD simulations were first applied to isolated hexamer-pentamer patches prior to global assembly onto a fullerene backbone and subsequent refinement. This early local relaxation allows capsomers to equilibrate toward local energetic minima before incorporation into the full lattice, which leads to more pronounced distortions of hexamers and pentamers across the structure.

More generally, deviations from an ideal flat hexagonal sheet arise because the capsid lattice must simultaneously satisfy multiple geometric and energetic constraints, including contributions from curvature, dimer and trimer interfaces, and the need for closure into a conical morphology. While regular hexamers are energetically favourable locally, our results suggest that the distribution of hexamer distortion is influenced by how capsomer interactions contribute to the fully assembled structure, and how relaxation dynamics is applied. In physiological settings, additional factors – including interactions with the genome and pH values– may further modulate these distortions.

3. In Ref. 28, triangular gaps arise specifically along the seam as a consequence of lattice mismatch, while the remainder of the capsid is well described by a hexameric lattice except near pentamers. Do the authors observe triangular gaps or analogous geometric features away from seam regions in the present data-derived lattice, or are such features likewise localized primarily near seams and/or pentamers?

Thank you for this important question. As clarified in our response to your Question 1 and in the revised Fig. 1, the mismatch regions in the data-derived lattice are not limited to triangular features. Instead, geometric discrepancies arise at both trimer and dimer interfaces, due to local rotations and positional adjustments of hexamers and pentamers relative to an idealised lattice.

Consistent with Ref. 28 (now Ref. 24), our analysis indicates that the most pronounced geometric mismatch is concentrated near the potential “seam regions”. This is evident from the spatial organisation of the geometric indices on the data-derived lattice, which form sheet-like domains that intersect at possible seam lines in the middle of the cone. Furthermore, our molecular frustration analysis shows that dimer interfaces near seams exhibit higher frustration levels and fewer H9 involving interactions, supporting the interpretation that these regions correspond to enhanced lattice mismatch.

4. The authors state that “typically, two to three seams appear in the bulk of each cone, joining adjacent sheets.” In Ref. 28, cones typically exhibit a single seam, and some cones grow without seams altogether. Can the authors’ model justify the presence of multiple seams energetically, given that seams are expected to carry an energetic cost? In addition, experimental work (e.g., Ganser and collaborators) has reported cones with different pentamer distributions (e.g., 5–7, 3–9,….). Have the authors considered these scenarios?

Thank you for raising these important points. First, we would like to clarify that **the observation of two to three seams refers specifically to the computational carbon fullerene cones** generated and optimised in the geometric and energetic modelling framework (Fig. 2d), rather than to biological HIV-1 capsids. By contrast, **in the reconstructed HIV-1 capsid *vlp23* analysed in this work, we observe a single dominant seam in the elongated region of the cone** (Fig. 4d; Fig. 6e-f), consistent with previous studies, including Ref. 28 (now Ref. 24). We have revised the manuscript to make this distinction clearer.

Regarding the energetic implications, seams indeed introduce local geometric mismatch and are therefore expected to carry an energetic cost. In the generated carbon fullerene cones, multiple seams arise because the Fullerene Program explores admissible geometric configurations without imposing biological or chemical constraints on assembly. These seams can be interpreted as boundaries between locally aligned sheet-like regions that minimise distortion within each region while accommodating global curvature. In biological capsids, assembly pathways and protein interactions likely bias the system toward configurations with fewer seams, possibly single-seam or seam-free cones. A similar phenomenon might occur for Carbon fullerene structures, favouring geometries with fewer seam lines over others.

Concerning the alternative **pentamer distributions** (e.g., 5-7, 3-9, …) reported experimentally by Ganser and colleagues, our current analysis focuses on atomistic structures that are presently available at sufficient resolutions. We agree that different pentamer arrangements are highly relevant and may influence seam formation. As additional high-resolution structures become available, it will be interesting to applying our geometric framework to these variants, which will be possible for any structure of interest given the methodology and associated computational routines provided here. We have added this point in the Discussion section.

5. Given that seam-like features can arise transiently or persist in cylindrical assemblies depending on growth pathways (e.g., Ref. 28), it would be helpful if the authors clarified whether the absence of seams in their cylindrical models reflects the specific construction and relaxation protocols used, and whether their geometric framework would be able to detect seam-like features in kinetically assembled cylinders.

Thank you for this important point. The cylindrical model (PDB: 6X63) analysed in this study corresponds to experimentally derived CA tubular assemblies that exhibit regular helical symmetry (-12, 11). As a result, their geometric organisation is dominated by parallel geometric index configurations (types 1-3 in Fig. 6d), and no seam-like discontinuities are detected. We therefore interpret the apparent absence

of seams as reflecting both the intrinsic symmetry of the reconstructed structures and the construction and relaxation protocols used to generate them. We agree that, as shown in Ref. 28 (now Ref. 24), seam-like features can emerge transiently or persistently depending on assembly pathways and kinetic constraints.

Importantly, the geometric framework introduced here is designed to identify discontinuities in the local lattice organisation through changes in both the magnitude and orientation of the geometric indices. Consequently, seam-like features – whether transient or persistent – would be expected to manifest as boundaries between regions with different local geometric index orientations, analogous to those observed in the conical capsid (*vlp23*) analysed in this work. We therefore anticipate that our method would be capable of detecting seams in kinetically assembled cylindrical systems when appropriate structural data are available.

A systematic investigation of seam formation and persistence in cylindrical assemblies generated under different growth pathways would indeed be valuable. Extending the current framework to such systems represents an important direction for future work, particularly when applying our geometric analysis across a wider range of experimentally observed capsid morphologies, both in virology and protein nanotechnology. We have made a corresponding addition in the Discussion section.

6. Instead of (or in addition to) the length of the shortest edge of the characteristic triangle, did the authors consider alternative geometric measures, such as a strain-like quantity directly related to elastic moduli (e.g., Young's modulus)? Such a metric might offer a more direct mechanical interpretation and could be worth discussing, even if only briefly.

Thank you for this valuable suggestion. In the present work, we chose the length of the shortest edge of the characteristic triangle as the primary geometric descriptor because it provides a simple, local, and model-independent measure of lattice distortion that can be robustly computed across different capsid geometries. Importantly, this quantity directly reflects the direction and magnitude of local compression within the lattice, and we show that it correlates with curvature, cone energetics, and molecular frustration. Our aim here was therefore to establish a minimal geometric metric that characterises structural organisation without introducing additional mechanical assumptions.

We agree that alternative measures – such as strain-like quantities or parameters more directly related to elastic moduli – could provide a more explicit mechanical interpretation. Prior work (Ref. 24, now Ref. 20, Deshpande, A. *et al. PLoS Pathog*) has explored capsid elasticity using molecular dynamics simulations, and a link between the distribution of residual stresses and capsid organisation has been provided in spherical viruses (https://pmc.ncbi.nlm.nih.gov/articles/PMC7482553/pdf/rsif20200455.pdf, [6],[8] inside), demonstrating that tangential shear stress is particularly important for viruses in the PRD1-adenovirus lineage. As we now highlight in the Discussion section, establishing quantitative links between lattice geometry and mechanical parameters therefore represents an important and promising direction for future research.

We have therefore added a brief discussion in the revised manuscript highlighting the potential extension of the framework toward elasticity-based metrics and mechanical interpretations in future studies.

7. When discussing curvature, do the authors primarily refer to angles defined between characteristic triangles, or to dihedral angles between neighboring capsomers? In Ref. 28, differences between cones and cylinders arise from differences in dihedral angles, and more recent kinetic studies of icosahedral assembly (Science Advances, 11, eady7241 (2025) have shown that changes in dihedral angles can alter assembly pathways (e.g., T=3 vs T=4). Clarifying how curvature is defined and interpreted here would help connect the present framework to these earlier results.

Thank you for this insightful comment. In the present work, when discussing curvature on the capsid surface, we primarily refer to **angles defined between the normal vectors of neighbouring characteristic triangles** located at local threefold axes. These quantities are conceptually related to dihedral angles between neighbouring capsomers, in that both describe local lattice bending, but they are not identical. Each characteristic triangle in our framework is constructed from three neighbouring capsomers and therefore provides a coarse-grained geometric measure of local surface curvature that integrates contributions from multiple protein-protein interfaces rather than focusing on a single capsomer-capsomer dihedral.

If the surface were fully tessellated into triangles (as in the icosahedral assembly work, *Science Advances*, 11, eady7241 (2025)), the resulting representation of the HIV-1 lattice would resemble a Kagome-like tiling. Instead, our approach uses triangles as local probes embedded within the native gyrated hexamer-pentamer lattice, allowing curvature to be quantified without redefining the underlying topology. For tubular assemblies, we further subdivided hexagons of the data-derived lattice into triangular elements and used covariance-based analyses to quantify angular relationships and curvature patterns (see Supplementary Fig. 11-12).

We agree that dihedral angles between neighbouring capsomers play an important role in determining morphology and assembly pathways, as shown in Ref. 28 (now Ref. 24) and the recent kinetic studies. The curvature metric introduced here can be viewed as an average over dihedral angle contributions in a local patch of the surface lattice around the characteristic triangle, and in this sense is related to it, capturing the cumulative bending contributions over this patch. We have now cited the study mentioned in the reviewer’s comments and added clarification in the revised manuscript to better connect our framework with prior dihedral-based interpretations.

Reviewer #2 (Remarks to the Author):

Twarock Revisión:

Li and coworkers present a study arguing that the geometry of the HIV-1 capsid is more accurately captured by a data-derived pseudo-tiling arrangement of hexamers

and pentamers, rather than by the idealized fullerene lattice traditionally used to describe it.

To support this hypothesis, the authors introduce a local geometric criterion to quantify deviations from an ideal fullerene lattice and show that these deviations correlate with specific biophysical properties of the capsid. They further suggest that such geometric deviations, and their associated biophysical features, provide insight into functional aspects of the capsid such as cofactor binding.

I consider the article is very interesting, the results are novel and I really enjoyed learning more about viruses and their properties while revising it.

Minor Comments:

Introduction:
In line 41 it says that there are 200 hexamers and 12 pentamers and that in many spherical viral shells there are deviations from the idealised lattices. In lines 60/61 it says that there is an imperfect alignment between adjacent hexamers / pentamers. I kind of understand the flow, but it is not clear that this observation comes from the atomic model derived from experiments in Fig1A, neither the panels are referenced but just Fig1 in general. I would ask authors to clarify so it is clearer and to reference individual panels in Fig1 to provide examples of each case.

Thank you for your suggestion. We have updated Fig.1 including the legend, and also revised the text to reference the specific panels in Fig. 1 where appropriate (Page 3).

Fig1a is mentioned later when they introduce that CryoEM has advanced… in my opinion it feels like the introduction comes forward and backward in the logical flow. It is understandable but a bit confusing.

Thank you for your advice. We now introduce Fig. 1a earlier (Page 3) and avoid re-mentioning it in the later paragraph (Page 4). We hope that this reorganisation will aid clarity of the exposition.

Line 68. I would write “Here in this work, we demonstrate…” to make it clear that it is this work and not past work from the authors. Also the word demonstrate sounds a bit too strong in this context.

Thank you for your advice. We’ve revised the sentence and replaced “demonstrate” as requested (Page 4).

You mention the hexamers and pentamers but no protein structures are shown in detail. I would like to maybe see them as an extra column in Fig1? 1 Hexamer and 1 Pentamer arrangement? Only the Hexamer appears in Figs 3 and 6.

Thank you for your suggestion. We’ve updated Fig. 1 according to your suggestion (Page 3 Fig. 1).

Fig2d legend: jointed -> joint.

Thank you, done.

Fig3: There are two lattices per panel. Are they rotations I guess (as they mention opposite sites of the structures in line 218), but not sure. Not described in the legend.

Thank you for pointing this out. You are correct that the presence of two lattice views per panel reflects rotated views of the same structure, corresponding to opposite sides of the capsid. We have added explicit labels indicating the rotational orientations and degrees in Fig. 3, as well as in other figures where structural rotations are shown.

Results:

Fig2: It is not clear to me from the text nor from the figure legend what are the 3 structures in panel d. Are they the same structures as in Fig1a with the geometric indexes and seam lines on top or are these rotations of the fullerene cone (as mentioned in the legend? the other structures/models are not mentioned and from there my confusion). In general, labels in the panels are easier to follow rather than having to read the legends.

Thank you for pointing this out. We have added short titles and labels directly within the panels of Fig. 1 and 2 and expanded the legends to explicitly distinguish between computational carbon fullerene models (Fig. 2) and HIV-1 capsid lattice representations (Fig. 1). Specifically, the structures in Fig. 1b represent the fullerene lattice representations derived from the HIV-1 capsid shown in Fig. 1a. The three structures shown in Fig. 2d correspond to rotated views of a single carbon fullerene cone example with geometric indices and seam lines indicated. We started our analysis with carbon fullerenes because the number of available data on HIV-1 capsids that are reconstructed to atomistic detail is currently limited. Fullerene models provide a large ensemble of structures that share key geometric features with HIV-1 capsids and have previously been used as backbones for HIV-1 capsid reconstruction (e.g., Ref. 16, Zhao, G. et al., *Nature*, 2013). This allows us to test the geometric index in a statistically broader setting before applying it to biological systems.

Line 219 the authors use “demonstrate” again. This is maybe personal but to show something is different from demonstrating.

We have now changed it to the word “show”.

Major Comments:

The authors analyse the binding of CypA and LEN. As CypA is a protein, can the authors analyse frustration changes upon binding between the lattice proteins and the CypA? The authors also say that binding at the CypA site induces local increase of the curvature whereas the opposite effect is observed when binding at the FG-

motif pocket. Does it correlate with frustration differences in those sites and the curvature change?

Thank you for this important suggestion. We agree that analysing the molecular frustration changes associated with cofactor binding and interfacial curvature changes would provide valuable physical insight. In the revised manuscript, we have therefore performed additional analyses to quantify frustration changes both at the binding interfaces and at the adjacent capsomer dimer interfaces before and after ligand binding (Pages 23-26). These results are presented in the new Fig. 8 and Supplementary Figs. 15-17.

Specifically, we analysed the interactions between CA and CypA and between CA and CPSF6 and observed that both CypA and CPSF6 binding reduces frustration at the binding sites, as expected. Importantly, we also found that CypA binding leads to an increase in frustration at the nearby dimer interfaces, whereas binding at the FG-motif pocket (by CPSF6) produces the opposite effect (reduced frustration at the dimer interfaces). These contrasting responses in frustration are consistent with the geometric changes observed in the study: CypA binding is associated with local curvature enhancement, while FG-motif pocket binding induces lattice flattening. We further validated the geometric interpretation by independently measuring local curvature changes before and after binding (Supplementary Fig. 15), which showed curvature increases upon CypA binding and decreases upon CPSF6 or LEN binding, in agreement with the geometric index predictions.

Taken together, these results demonstrate a correlation between changes of frustration levels at the dimer interfaces and curvature changes, implying that changes in frustration can be indicators of curvature changes.

In the section “Correlation between capsid topography and molecular frustration at dimer interfaces”

The authors say “we extended the concept of molecular frustration from the Protein Frustratometer…”. I disagree with this wording. What the authors are doing is to calculate the average frustration per contact involving the h9 helices at the interfaces. That is not, in my opinion, a conceptual extension but an application. The authors are analysing the frustration patterns as calculated by frustratometer at the interfaces. I suggest rewriting this part.

Thank you for your comment, we haven rewritten this part as you suggested (Pages 11-12).

In this same section, the authors refer to frustration “levels”, whereas typically we refer to frustration “states”. Also, the authors change the frustration index sign which is confusing regarding how frustration indexes are interpreted in the literature where high frustration is denoted as negative Z-scores compared to the decoys and minimal frustration as positive. Changing the sign of the indexes could confuse readers and I do not see this is needed to make the analysis and interpretations the authors are making.

Thank you for your clarification. We agree that the terminology and sign convention should follow established usage in the frustration literature.

In the previous version, we had introduced an inverted sign so that larger numerical values correspond to higher frustration. However, we agree that maintaining the conventional definition in keeping with the literature is preferable. The Methods and figure legends have been updated accordingly.

In the same section the authors could cite the papers where frustration is correlated with the functional location of binding sites (ferreiro et al pnas 2007, parra et al plos comp biol 2015) and allosterism (ferreiro et al pnas 2011).

Thank you for your recommendation, we have now cited these important works, as they are highly relevant to the correlation of frustration states with the functional location of binding sites.

The authors also mention: "In the original approach, the frustration level of a residue is derived from the proportion…". This statement is inaccurate and may mislead readers unfamiliar with the Frustratometer methodology.. Frustration states of residues/contacts are based on the comparison of the total energy change between the native protein and the decoys. There is another secondary analysis Frustratometer returns that is the proportion of contacts classified in each frustration state so the contact frustration indexes (configurational and mutational) can be assessed at single residues, which complements the single residue frustration index that can mask bimodal distributions of states. Such analysis counts the proportion of contacts of each state in a sphere of 5 Angstroms. In that sense, I guess we could say their analysis is somehow analogous. Still, I think the authors should rephrase to be precise about these issues.

Thank you for pointing this out, we have now taken your suggestion into account and rephrased the statements to be more precise (Pages 11-12).

The results shown in Fig4 and movies 1, 2 and 3 are very interesting. In frustration analysis we have 3 types of contacts: short, long and water mediated. We usually show water mediated interactions in dashed to differentiate them from the short and long. In the movies everything is shown in dashed lines (I guess for simplicity) so I cannot differentiate the water mediated from the non water mediated contacts. The different contact types (the classification is available in the frustratometer output) use slightly different hamiltonians based on distance and solvent accessibility. It would be interesting to observe if the longer distances between interacting proteins correlate with solvent accessibility changes and changes in overall frustration. If yes, this may point out that the geometric index and changes in curvature modulate function by changing solvation at the interfaces which is translated in frustration differences between models and so on.

Thank you for this insightful comment. We agree that distinguishing contact types in the visualisations is important. In the revised movies, we have updated the representations so that water-mediated interactions are shown as dashed lines, whereas direct contacts are shown as solid lines, consistent with the standard

convention used in frustration analysis. This should make the different interaction types easier to interpret.

Following your suggestion, we also performed additional analyses to examine whether interfacial geometry correlates with solvent-mediated interactions and overall frustration. We observed that interfaces with larger separations at the dimer interfaces exhibit a higher proportion of water-mediated contacts and higher frustration, whereas interfaces with tighter packing contain more direct contacts and lower frustration. This relationship is now quantified in Supplementary Fig. 7c. Furthermore, to assess whether the observed seam-frustration correlation depends on solvent-mediated interactions, we repeated the analyses excluding all water-mediated contacts. The spatial localisation of highly frustrated interfaces along the seam side remained unchanged, and the correlations with contact number were preserved (see Supplementary Fig. 8; Pages 11-14).

We thank the reviewer for this suggestion, which has strengthened the mechanistic interpretation of our findings.

In Fig4f I got confused. What is the frustration “level” that is being plotted? Is their averaged frustration value per interface contact involving h9 helices and the number of contacts being considered in such calculation? Basically, when there are less contacts in the interface they are also more highly frustrated on average? (I had to see these multiple times as the change in sign made me conclude the opposite, see previous comments about consistency with previous frustration analysis) In that panel, I would be a bit cautious. The number of interface contacts (y axis) ranges from ~200 to ~260, while the mean frustration changes (x-axis) by ~0.5 units and remains largely within the neutral regime. While the trend is clear, additional analysis (e.g., proportions of highly/minimally frustrated contacts) would strengthen the interpretation. The effect is there, I am not doubting it, but to make it stronger I think a bit of analysis of proportions would help. In our experience averaging always takes one to the neutral region. That is why using discrete states as proportions usually helps. The authors could analyse to put on the x axis the proportion of interface contacts being actually red, gray or green. Are more dense interfaces (shorter gap) harbouring more red contacts? I think it might be true that interfaces that are more solvated lead to less contacts (because of distance) and the ones that remain as they are water mediated are more frustrated. When distances become shorter, they become energetically more optimized. But the authors should analyse this more carefully and see if this is true. From panels a,b,c I am not sure if there are more highly frustrated interactions but more neutral instead when the gap is larger. When the gap becomes shorter there are more minimally frustrated contacts. This could be enough to explain the effect, very interesting if true. It would be useful to assess whether the co-localization of frustration and seam lines is robust to alternative frustration aggregation schemes (e.g., median frustration, weighted by contact area, or excluding water-mediated contacts), as averaging may mask heterogeneous distributions.

Thank you for your suggestions. The quantity plotted in Fig. 4f corresponds to the correlation between the average configurational frustration index per dimer interface, computed across all contacts involving the H9 helices (x-axis) and the number of contacts representing the total interfacial interactions included in the computation (y-

axis). We have clarified this definition in the revised manuscript and restored the standard sign convention to avoid confusion.

Following your suggestion, we performed additional analyses quantifying the proportions of highly, neutral, and minimally frustrated contacts as a function of interface contact number (Supplementary Fig. 7d-f; 8c-e). These results confirm that interfaces with fewer contacts contain more highly frustrated interactions, whereas tighter interfaces contain more minimally frustrated interactions. We also analysed the relationship with water-mediated contacts (Supplementary Fig. 7c) and repeated the mapping after excluding water-mediated interactions (Supplementary Fig. 8), showing that the co-localisation of highly frustrated interfaces with the seam side remains robust (Pages 11-14).

I am not sure what the authors want to say in lines 263-264: “Strikingly, ..”.

We have rephrased this sentence to more explicitly describe the intended observation, which is the spatial co-localisation between highly frustrated dimer interfaces and the seam side defined by the geometric index.

The co-location of frustration with the seam line is a very compelling result that could explain the functional modulation of the capsid.

All over the manuscript, there is no consistent mention to the models. Sometimes they are named as idealised, data derived, etc.. whereas in other cases are mentioned by their pdb ids or other nomenclature. I think the authors have to refer to the different models and lattices in a consistent way so it is easy to keep the logic through sections. It is especially difficult when PDB IDs are used. The authors are really familiar with them but for non related readers those IDs mean nothing and it is difficult to connect/remember which one is which.

We agree that consistent terminology is important to maintain clarity throughout the manuscript.

The idealised and data-derived lattices represent the geometric lattice representations of the HIV-1 capsid, whereas the structures referred to by PDB IDs correspond to the atomistic models deposited in the Protein Data Bank. We have revised our text to better distinguish geometric representations from atomic structures, but we think it is important to keep the PDB identifiers when referring to specific structures, as this is standard practice in the field.

We agree that this may be less intuitive for the general readers; however, replacing PDB IDs with alternative names could introduce confusion for readers familiar with these structures, similar to the issue encountered when we previously modified the frustration index convention.

I wonder if authors could make a big matrix of pairwise correlations of: number of interface contacts, averaged frustration per interface contacts, geometric index, curvature factors phi1 and phi2, etc. Because are the values in Fig6c correlated with number of contacts and frustration? If yes, how would it be interpreted? I guess this is related to the sentence in lines 373-375. To observe those correlations would

strengthen the claim.

Thank you for your suggestion. The geometric index and curvature factors (phi1 and phi2) are defined around the threefold axes based on the characteristic triangles, which differs from the dimer interfaces where the molecular frustration values are computed due to their importance in capsid formation, so that direct pairwise correlations are not straightforward to interpret. The interfaces between triangular units in Fig. 6d correspond to the different orientations that the geometric indices can take on in neighbouring characteristic triangles, and should be interpreted as the orientations of local strains. As these interfaces are co-located with the dimer interfaces at the particle 2-fold axes they can be shown superimposed, as is done in Fig. 6e&f.

Line 377: “universal”. Sounds too strong. Do you mean it could be used to describe any other capsid from any other virus? I would use such a word if a very large set of case studies would be analysed with this.

We have removed the word.

In Fig7 the authors show binding of CypA on top of the lattice arrangements. Did the authors quantify frustration in the CypA-bound state and CypA and compare if the complex leads to less frustration when quantified in the complex vs in the monomers alone? Release of frustration upon binding, even with this artificial way would be a good support to their results.

Thank you for this suggestion. We agree that comparing frustration between the bound complex and the isolated components could provide additional insight into binding energetics. However, an accurate comparison would require structures of free CypA under conditions comparable to those in the bound complexes, for which structural data are currently not available. Simply removing CypA from the complex would introduce structural artifacts, because the conformation of CypA in the bound state is already adapted to the interaction interface, leading to potentially biased frustration estimates. Instead, in the revised manuscript we quantified frustration changes before and after binding within the CA lattice context, analysing both the binding regions and adjacent dimer interfaces.

Methods:

By checking the supplementary material I see that the authors have used the configurational frustration index. Which is good as it correlates better with protein-protein interfaces. However, the authors do not mention that the configurational index is used. Please do so readers know.

Thank you for your suggestion, we have precisely defined this in both the main text and the Methods section.

The authors say: “Local energetic frustration was quantified based on previous work”. I understand that the authors did not code their own algorithm to calculate frustration but used Frustratometer. So I think the correct wording would be “Local

energetic frustration was quantified using the Frustratometer tool (citations). The configurational frustration index was used as it has been shown that it better correlates with protein-protein binding sites (Ferreiro et al pnas 2007, parra et al plos comp biol 2015).

Thank you for this comment. We have rephrased the text accordingly. In our analyses, we used the Frustratometer's computational framework (LAMMPS-based implementation) to compute the configurational frustration index for protein-protein contacts. We then performed additional region-specific analyses and aggregation steps tailored to this study, as the standard Frustratometer outputs (e.g., residue-level state fractions) are not directly applicable to interface-level comparisons.

Summary of my revision:

I think the article by Li et al presents a very interesting way to link local geometries to higher order arrangements that in turn lead to specific frustration patterns in the lattices that may lead functional processes like the binding of co-factors.

The text is sometimes difficult to read due to vague or inaccurate phrasing and inconsistent use of terminology (at least in the case of frustration). Figures not having labels force readers to go back and forth between them and legends' difficult interpretation. Specifically, the way they refer to the different lattice geometries took me a lot of time to make sense of the data.

That being said, I do think that the main results are supported by their analysis. I have proposed some complementary analysis that in my opinion would strengthen their claims.

To me the most interesting part is that according to their data, the distorted lattice geometry that has more frustrated interactions on average (and broader gaps) would not be my first choice between models because one would expect protein-protein interactions to be minimally frustrated in a well formed capsid. The fact that the model supported by the data has the border gap with more frustration suggests a functional requirement, maybe to bind the co-factors. That is not discussed as such in the discussion or anywhere in the text.. maybe it is not true, but the authors have the data to prove it and if true, that is REALLY interesting, at least to me.

It is also surprising to me that the authors only briefly mention their work in reference 40 that has nicely shown how local frustration can help orchestrate virus assemblies.

We thank the reviewer for the thoughtful evaluation and for recognising the potential significance of linking local geometry, lattice organisation, and molecular frustration to functional processes such as cofactor binding.

In the revised version, we have incorporated the additional analyses suggested by the reviewer, including additional frustration and curvature comparisons, robustness tests, and proportion-based frustration analyses, which further strengthen the conclusions. We have also substantially improved clarity by standardising terminology (particularly for molecular frustration), adding labels directly within

figures, and clarifying the distinctions between the different lattice geometries throughout the text and legends.

Reviewer #3 (Remarks to the Author):

In the paper by Li et al, they define a new geometric metric, referred to as the geometric index, that reports a length scale on each 3-fold axis within a defined lattice. The key observation is that this index varies by lattice assigned to a structure: fullerene vs kagome vs data-derived, and for the data-derived lattices, it is a good predictor of lattice energy and curvature due to the correlations. The index also correlates with binding sites for small molecules.

The metric certainly seems useful, and the figures are generally clear about the geometric aspects, although several of the figure subplots with numerical data are hard to read.

We have reorganised the figures to improve readability. We hope these revisions make the geometric and quantitative comparisons clearer to the reader.

It is unexpected that the definition of the lattice topology (e.g. fullerene vs data-derived) which primarily differ in what angle the hexagons adopt relative to one another, is more or less informative about the lattice properties. The comparison of the topologies is also interesting, that drawing boundaries between hexagons in distinct ways can be more or less informative about the lattice properties. Frustration between dimers, for example, is correlated with the data-derived lattice seams. Overall, the geometric metric, if it is easy enough to calculate from an experimental structure, seems to be an informative way to visualize the structure and assess where frustration may exist in the lattice. It is harder to tell how predictive it will be.

A limitation is that the results of the geometric index seem to be correlative, rather than causative or truly predictive. The authors did not use the index to test a hypothesis about is predictive power, by, for example, designing a structure to have specific properties, whether of energetics, frustration, or binding.

We agree that our previous version has not been explicit about this point. We have now performed additional analysis in which we clearly formulate a hypothesis regarding the effect of co-factor binding on local curvature and frustration states that we are validating with our formalism. We hope that this additional work added in the revised version (see pages 23-26, new Fig. 8 and also Supplementary Fig. 15-17) illustrates the way in which our geometric index can be used to predict biophysical quantities of interest.

In addition, we would like to stress that our theory predicts the locations of seam lines, which is of interest for understanding the weak point of the capsid and thus potential areas of rupture and genome/cargo release.

It is also not clear to me how many distinct HIV capsid lattices were actually evaluated, and therefore how robust the trends are (what is N?) I think it is 3, vlp23 and two PDB structures, but this is not clearly stated.

Any such assessment is currently limited by the number of available data sets. This is why we validate our theory regarding seam line prediction first on the larger ensemble of computationally generated Carbon fullerene cones (654 structures). In a second step, we evaluated three distinct atomistic HIV-1 capsid reconstructions generated using different methodologies: the *vlp23* capsid and two experimentally derived capsids (PDB: 3J3Y and 3J3Q). These systems were selected because they represent currently available atomistic reconstructions with sufficient resolution and differing assembly protocols.

Major comments:
1. What are the C524, etc, datasets? Is this one single capsid lattice? From the SI it looks like maybe 3 lattices were studied? They mention Carbon atoms repeatedly, but the subunits of the capsid lattice are complete proteins, so I don't understand this. Are the 654 fullerene structures each a different experimental lattice? Why are there such specific numbers (325, 452) of vertices? What is vlp23, a single structure?

Thank you for these questions. The datasets denoted as **C352, C452, and C524** refer to **computationally generated carbon fullerene cones** containing 352, 452, and 524 vertices (carbon atoms), respectively. These structures were generated using a fullerene construction program, in which different cone geometries arise from varying the positions of pentagons at the two ends of the cone, followed by energy optimisation (see Methods: Computational Fullerene and Quantum Mechanical Methodology). Each individual fullerene within a dataset therefore represents a distinct geometric realisation of a cone with the same total number of vertices but different pentamer arrangements. The specific vertex numbers arise from topological constraints of fullerene cones and correspond to different cone sizes and morphologies. These are not experimentally obtained HIV-1 capsid lattices, but idealised geometric models used to establish general relationships between lattice organisation, geometric index, and cone energetics.

We began with carbon fullerenes because the number of currently available HIV-1 capsids that are reconstructed at atomistic scale is currently limited. Fullerene models provide a large ensemble of structures that share key geometric features with HIV-1 capsids and have previously been used as backbones for HIV-1 capsid reconstruction (e.g., Ref. 16, Zhao, G. et al., *Nature*, 2013). This allows us to test the geometric index in a statistically broader setting before applying it to biological systems.

The biological analyses in this study focus on **three independent and complete HIV-1 capsids**: the ***vlp23*** capsid and two experimentally derived capsids (PDB: **3J3Y** and **3J3Q**). The *vlp23* capsid is a fully reconstructed atomistic HIV-1 capsid model derived from cryo-EM data following molecular dynamics refinement. We have now added explicit labels in Fig.1 and 2 and revised some text and legends to clearly distinguish between the computationally generated carbon fullerene models and the HIV-1 capsid structures, to avoid ambiguity.

2. In Figure 2f, for geometric index vs energy, I'm not quite sure what energy is being reported—the range is very small, from -9.685 to -9.702, is this significant? What is each dot in the plot from?

Thank you for your questions. Each dot in Fig. 2f represents **a single computationally generated carbon fullerene cone from the C524 dataset**, which contains cones with 524 vertices (carbon atoms). In total, 290 distinct fullerene structures with the same number of vertices but different pentagon arrangements and morphologies were analysed and are represented by the 290 dots in Fig. 2f. The **x-axis shows the sum of the geometric indices for each cone**, $\sigma = \sum_{i \in I} \lambda_i$ where $\lambda_i := \min_{j \in \{1, 2, 3\}} l_{ij}$ (Equation 1), while the **y-axis shows the intrinsic binding energy per atom (E_b)** obtained after energetic optimisation using the quantum mechanical methodology described in the Methods section.

Although the numerical range of energies (approximately -9.685 eV to -9.702 eV) appears small, this variation is meaningful because **all structures contain the same number of atoms and differ only in their geometric organisation**. In this context, even small per-atom energy differences correspond to measurable differences in relative conical energetic preference and reflect subtle but systematic changes in lattice strain. The observed correlation therefore indicates that the geometric index captures physically relevant aspects of the underlying lattice energetics.

3. For Figure 5, they are comparing lattices that were constructed using distinct protocols during the cryoEM reconstruction. I am not sure what to conclude. Are the authors suggesting that the atomic structures fit to the lattices are more accurate depending on which protocol is used, or that the properties of the lattice will be different depending on how the atomic structure is fit to the cryoEM data?

Thank you for this important question. Our intention in Fig. 5 is not to suggest that one reconstruction protocol necessarily produces more accurate atomic structures than another. Rather, the purpose of this comparison is to demonstrate that **different reconstruction** (and potentially assembly) **procedures can lead to capsid models with distinct geometric organisations**, which are detectable using the geometric index and may influence inferred lattice properties.

Specifically, different procedures impose different geometric constraints during model building and energy relaxation. For example, in *vlp23*, the full capsid was assembled prior to global relaxation, whereas in the other two capsids (PDB: 3J3Y and 3J3Q), local hexamer-pentamer patches were equilibrated before global assembly. These differences can allow capsomers to equilibrate under different conditions, resulting in measurable variations in geometric index distributions, hexamer regularity, and overall lattice organisation (Fig. 5 and Supplementary Fig. 9-10).

Therefore, our conclusion is that **the geometric and biophysical properties of the lattice are intrinsically coupled, and both depend on how the structure is reconstructed** (or assembled), rather than making a statement about absolute accuracy. In this sense, the observed differences may also reflect distinct assembly

histories or kinetic trajectories, which the geometric index can help to quantify in future studies.

4. Following this, the authors mention that the geometric index can report on assembly pathways. This would be interesting, but is fairly indirect given that the data are based on reconstruction methods rather than dynamic assembly trajectories.

Thank you for this helpful comment. We agree that the inference of assembly pathways in the present work is necessarily indirect, because our analysis is based on reconstructed static structures rather than explicit dynamic assembly trajectories.

Our intention was not to claim that the geometric index can directly determine assembly pathways. Rather, we show that the geometric index captures systematic differences in lattice organisation that arise from distinct reconstruction workflows, which impose different geometric constraints during model building and relaxation. These differences are consistent with the idea that assembly history leaves measurable geometric imprints on the final lattice. In this sense, the geometric index provides a quantitative descriptor that may help distinguish structures formed under different assembly conditions or kinetic regimes, rather than reconstructing the pathways themselves.

We therefore view this aspect as a hypothesis-generating observation: if assembly pathways influence how capsomers equilibrate and pack, then corresponding geometric signatures should be detectable in the final structures. Direct validation would require time-resolved simulations or experimental assembly trajectories, which are beyond the scope of the current study but represent an important direction of future work.

We have further clarified this point in the revised manuscript to avoid overinterpretation and to emphasize that our conclusions regarding assembly pathways are interpretive rather than definitive.

5. The analysis of the CypA binding and the LEN binding is a bit hard to follow. Binding of the cofactors are reported as correlated with the geometric index, but I don't see this from Fig 7 data. I can't tell what is analysis of the known data, and what is the prediction.

Thank you for this comment. To the best of our knowledge, there is currently no study that systematically characterises the geometric features and responses associated with different cofactor and small-molecule binding sites on the HIV-1 capsid, nor analyses how such binding influences molecular frustration at key capsomer interfaces. Addressing these questions is one of the primary goals of the final two sections of this work.

We have significantly expanded this section in the revised version and have formulated a hypothesis that we validate. We hope that this makes the message clearer to the reader.

In Fig. 7 (and Supplementary Fig. 11), our analysis consists of several conceptually distinct steps:

1. **Analysis of the known structures (observation).** We first analysed experimentally determined CypA-CA complexes with different helical symmetries (Fig. 7a, b; Supplementary Fig. 11). From these structures, we identified the geometric indices at the relevant threefold axes and observed a consistent geometric pattern: the CypA-binding monomer (cyan) lies opposite the shortest edge (the geometric index, cyan dashed line) of the characteristic triangle, and this geometric index is smaller than that of its neighbouring triangle (purple dashed line). The associated dimer interface corresponds to the type-2 configuration defined in Fig. 6. This is a conclusion from applying the geometric index to all available data sets on the PDB.
2. **Prediction on tubular assemblies.** Based on this consistent geometric pattern, we mapped the inferred geometric rules onto an independent CA tubular structure (PDB: 6X63) that does not include bound CypA (Fig. 7c). Using the geometric index alone, we identified candidate binding sites and subsequently positioned CypA molecules by structural alignment. The resulting model predicts that CypA preferentially bridges two CA hexamers along the direction of lowest curvature, consistent with previous experimental observations. This step represents the predictive component of the analysis.
3. **Prediction and validation of curvature changes upon binding.** For LEN and CPSF6 binding at the FG-motif (Supplementary Fig. 14), we compared structures before and after ligand binding and quantified changes in geometric indices, predicting opposite curvature response (lattice flattening) relative to the CypA site (curvature enhancement). In the revised manuscript, we added independent curvature measurements to validate these geometric index-based curvature change predictions (Supplementary Fig. 15).
4. **Coupling between binding, geometry, and molecular frustration.** In order to strengthen the link with the biophysical properties of the capsid, we performed more analysis for the revised version, now linking the geometric index not only with curvature changes due to ligand binding, but also with changes in molecular frustration. In particular, we further analysed molecular frustration changes at the CypA and CPSF6 binding sites and at the adjacent dimer interfaces before and after ligand binding (please see the new Fig. 8 and the new Supplementary Figs. 16-17). These analyses reveal ligand-specific frustration responses in line with the observed geometric changes and provide additional mechanistic insights into how cofactors influence capsid behaviour. We hope that this makes the link between the geometric index and changes in the molecular frustration upon ligand binding clearer. In particular, this link suggests that the geometric index can be used to identify changes in the molecular frustration profiles upon ligand binding, which in turn are important for understanding changes in the capsid (e.g. at the dimer interfaces as we show here) in response to ligand binding. The geometric index might be viewed as an indicator of such changes.

To improve clarity, we have added arrows, labels, and explanatory text to Fig. 7, introduced new Fig. 8 and Supplementary Fig. 15-17, and revised the manuscript to improve readability and avoid confusion.

6. The step of showing how the geometric index could be used in practice to design something with specific properties or select for stronger or weaker binding of co-factors is not pursued. This leaves the study as more speculative than truly demonstrative of its utility.

Our theory can be exploited in combination with machine learning techniques such as RFdiffusion (https://github.com/RosettaCommons/RFdiffusion), but this would be out of the scope of this paper. Briefly, our geometric index captures local variations from the idealised symmetric state of the lattice, which can be built as geometric constraints into such design protocols for oligomeric states. We now mention this in the Discussion to make the prospect of such applications more tangible.

REVIEWER COMMENTS

Reviewer #1 (Remarks to the Author):

The authors have revised the manuscript in response to the comments from the other referees and myself. I therefore recommend the paper for publication.

Thank you very much for your comments and suggestions that have helped us improve our manuscript.

Reviewer #2 (Remarks to the Author):

I am pleased that the authors have taken my suggestions seriously and have made a careful and thorough revision of the manuscript. I appreciate the clear effort made to address the majority of my comments, both in terms of improving the clarity of the presentation and, importantly, by incorporating additional analyses which I consider have made their article stronger.
In particular, the new results linking cofactor binding, the curvature changes, and molecular frustration (Fig. 8 and associated supplementary material) significantly strengthen the manuscript and provide a more compelling connection between geometry and biophysical function.
I also note that the authors have corrected important technical aspects regarding the use and interpretation of molecular frustration (terminology, sign conventions, and methodological descriptions), which improves the rigor and accessibility of the work.
The improvements in figures and labeling have also made the manuscript easier to follow.

That said, I have the following minor points and curiosities from my side:
The word demonstrate still remains in the abstract. I checked the authors have modified it in the main text.
Page 11. When the authors explain that they used the configurational frustration index, they use the word metric instead of index. The frustration index is a z-score and therefore not a metric which is a mathematical concept that satisfies very specific constraints. The correct word is index, not metric.
In page 17, they also use the word metric to refer to the curvature which would be more accurately referred to as “measures”, “scores” or any other word that better reflects what that quantity is.
Page 19. They also use the word metric to refer to the geometric “index”.

Thank you for your advice, we have corrected the words accordingly.

The analysis of frustration changes in the interfaces with CypA and CPSF6 is interesting. The change that is most noticeable is the delta in binding of CypA followed by the delta in the binding of CPSF6. For the interfaces, maybe the

averaging is masking some interesting results. First, it would be good to add the number of considered residues both at the binding sites and at the interfaces in both cases which I can kind of quantify from supp figs 16 and 17(add to Table 1). But also, you could include the mean 5 of virtual particles of each residue (you can find that data in the 5Adens output for the configurational index) that tells you the proportion of each frustration class in a 5 Angstroms sphere from each residue (e.g. the ones at the binding and interface residues in each case). This may unmask some interesting patterns (similar to the fig2 in this article from the last author: https://www.cell.com/trends/microbiology/fulltext/S0966-842X(23)00200-7).

Thank you for your comments. Following your recommendation, we have added additional columns in Table 1 to explicitly indicate the number of residues included in the binding regions and dimer interfaces in each case. More details as requested are given in the "Frustration Changes upon Cofactor Binding" part in Methods.

Regarding the frustration class proportions within a 5 Å neighbourhood, we carefully evaluated this suggestion. However, we found that it is too crude for comparing the subtle changes upon cofactor binding in our system. Specifically, many interactions undergo shifts in the configurational frustration index that do not cross the classification thresholds. For example, a contact may change from 0.5 to -0.5 (remaining neutral but becoming more frustrated), whereas another may shift from -0.99 to -1.01 and be reclassified as highly frustrated despite only a marginal change. As a result, categorical proportions can mask meaningful continuous changes, particularly when most contacts remain within the same frustration class.

In addition, the relatively small number of contacts within a 5 Å neighbourhood per residue limits the sensitivity of proportion-based measurements. In our analysis, observable changes in class proportions are confined to the residues directly involved in binding and largely reflect the expected reduction in frustration at the binding sites.

For these reasons, we chose to use the average configurational frustration index, as before, which provides greater sensitivity to continuous changes in interaction energetics between Apo and ligand-bound states. This approach allows us to capture subtle but systemic shifts that are not apparent from discrete class proportions.

The number differences in the table are relativelly small, in my opinion, both in the neutral range.. I would not overinterpret them unless a dissection as explained before throws some more meaningful result.

Thank you for your suggestion. In the revised manuscript, we have expanded the analysis in several ways. First, we included additional adjacent dimer interfaces in

both systems, now averaging over more residues than before. We have updated the corresponding frustration index values, taking into account this larger residue set (see Tab. 1&2). In the previous version, only a single adjacent interface was considered for the CypA-bound system, which led to relatively modest observed changes. The new table now reflects the systemic changes.

In Fig 16 I do not see a big difference at the interface of CypA. I do see a bigger difference for CPSF6. Is the binding of these proteins to the capsid triggering some sort of allosteric response? I think that is a very nice hypothesis that the authors have written in that section although I would say the allosteric effect is larger in CPSF6, if true :)

The referee is right – the effect for CypA at any given interface is small. However, CypA binding affects different interfaces. To reflect this, we have now averaged over the effect at all interfaces concerned (Tables 1&2). In this case, the effect for CypA binding and CPSF6 binding is more comparable. In any case, CypA binding occurs more distal from the interfaces than CPSF6 binding and this effect on the dimer interfaces could therefore be classed as allosteric; in the case of CPSF6 binding it is directly adjacent and in contact with the interface where the effect takes place, so it is not really allosteric in this case.

In the same page the authors say "while these observations highlight opposing geometric and frustration responses at the two primary binding sites". Again, the changes are small and both remain in the neutral region of frustration and also are measured in static complexes. I would not overinterpret the effect at the interfaces as "opposing" as they are energetically in the same class of frustration both after and before the binding. In addition, the authors could analyse the residues of each group (binding and interfaces) individually. The averaging may be masking some energetic signature because not all residues behave the same way. As assembly and disassembly are reversible processes when some residues reduce frustration after binding, some others may increase it (see https://www.nature.com/articles/s41586-025-09549-z) and vice versa. Therefore, the authors can analyse residues individually to see which are the residues that have the highest change and potentially drive the process while the others just follow or prone the complex to dissociation once bound.

With the expanded analysis, we find that prior to binding, the dimer interfaces in both systems exhibit similar frustration values. Upon binding, however, CypA induces an increase in interfacial frustration, whereas CPSF6 binding leads to a decrease. We have clarified these observations in the revised manuscript.

To further probe these effects, we complemented the average frustration analysis with proportion-based measurement over the cofactor binding region and interfacial

region (Table 2), considering all contacts within 5 Å of the residues in the binding regions and the adjacent interfaces. While residue-level proportions show minimal variation due to the limited number of contacts per residue, aggregating at the regional level reveals clearer trends, consistent with the geometric changes observed. These results support a qualitative distinction between the interfacial responses to CypA and CPSF6 binding.

Fig 8g and h. Unfortunately, the color of the protein structures (red and yellow) is very similar to the frustration lines (red and green) and it becomes difficult to see anything.I would play around with the protein structure colors to make it less difficult to observe.

We have revised the colour scheme accordingly to avoid confusion. In addition, due to the high abundance of neutral contacts, we have omitted them from the visualisation (as is done in other publications in this area) and now display only highly and minimally frustrated interactions to enhance clarity and facilitate comparison.

In summary, I think that the article is greatly improved and clarified. I just would like the authors to evaluate every instance where they use the words demonstrate and metric. I think that in most cases, if not all, the use of these terms is inaccurate. The message is clear, but accurate wording is clear and nicer.

Congratulations for such nice work that enriches the applicability of frustration analysis to complex systems. I do not consider it necessary to review the manuscript again.

We appreciate your insightful comments, which have helped us refine both the analysis and the interpretation.

Reviewer #3 (Remarks to the Author):

The authors have responded to my comments, the figures and the text are more clear, as are the predictive applications.

Thank you very much for all your questions and comments.

Reviewer #3 (Remarks on code availability):

The Zenodo repos do not appear to be active, I cannot find either doi, I also searched by paper title and did not find them.

The repositories were not published as the article had not been accepted at that time, but we provided a specific access link to the reviewers in the code checklist and reporting summary. They have now been published and are publicly available on the links given in the manuscript.
